# The Future of Pharmacogenomics Requires New Discoveries and Innovative Education

**DOI:** 10.3390/genes13091575

**Published:** 2022-09-02

**Authors:** Emiliano Giardina, Stefania Zampatti

**Affiliations:** 1 Genomic Medicine Laboratory UILDM, IRCCS Fondazione Santa Lucia, 00179 Rome, Italy; 2 Department of Biomedicine and Prevention, Tor Vergata University of Rome, 00133 Rome, Italy

Since the beginning of pharmacology, several variations in responses to drugs have been recorded. Pharmacodynamics and pharmacokinetics show many interindividual differences, that can be acquired or inherited [1]. Pharmacogenomics is a field of genomics focused on the study of genomic interindividual variations and their relationship with different responses to drugs. The availability of a new generation of technologies for genomic characterization of subjects led to an incredible increase of knowledge in pharmacogenomics. Unfortunately, this huge amount of genomic data is not immediately translatable in clinical practice. Several studies are generally needed to verify, confirm, and applicate pharmacogenomic data in clinical practice. Personalized medicine takes advantage of these data to define medical models that are useful to tailor the right therapeutic strategy to the right patient at the right time. To date, a range of pharmacogenomic evidence have been validated and included in guidelines to individualize treatments, optimize dosage, and prevent adverse effects [2,3]. Despite known ethnical differences in genotype frequencies across populations, some interesting associations have been reported. In this Special Issue, Fernandes et al. described how genetic ancestry can significantly alter the response to drugs [4]. Studying children in the Amazon region of Brazil, authors investigated possible associations between *NUDT15* (rs1272632214) and *SLC22A1* (rs202220802) gene polymorphisms and response to treatment (BFM 2009 protocol) in acute lymphoid leukemia patients. Another myeloproliferative neoplasm, chronic myeloid leukemia (CML), and its first-line treatment imatinib are investigated by Cereja Pantoja et al., who conducted a large genotype association study on 32 polymorphisms in several carcinogenic genes, revealing interesting results [5]. To date, many oncological treatments recognize pharmacogenomic therapeutical suggestions. Franczyk et al. reviewed polymorphic variants associated to therapeutic differences in individual response to anticancer drugs [6]. FDA-approved biomarkers for anti-cancer drugs and polymorphic variants with evidence of pharmacogenetic association are reported in this review, showing available application in clinical practice and promising research areas. Similarly, Caputo et al. summarized potential genetic biomarkers in biological and new-generation drugs for psoriasis treatment [7]. Genetic polymorphic variants and their reported association with different drug pathways and individualized response to treatments are described. A practical approach is offered by Marucci et al., in their review on monogenic diabetes [8]. Different genetic forms of diabetes are reported, and available treatments are described. “Actionable genes” are listed, with their molecular and physiopathological mechanisms of disease. The focus on the actionability of each phenotype provides a practical vision of genetic disorders, outlining potential strategies for personalized medicine.

The individualized genomic response to treatment is driven by the huge amount of genetic inter-individual variants. This variability firstly acts at a cellular and molecular level. The study of Guan and coworkers reports molecular patterns that underlie the survival of prostate cancer cells. The study is based on previous findings about the recruitment of tumor-associated macrophages (TAM) mediated by CSF-1 secreted by docetaxel-treated prostate cancer cells [9]. In this paper, authors reported the role of CXCL12 and CXCR-4 in drug resistance and the survival of cancer cells.

The increasing evolution of genomic technologies permits the introduction of the rapid genomic assessment of patients in clinical practice. Despite numerous applications of genomics in diagnostic protocols, pharmacogenomics involves many genes and even more numerous polymorphic variants. Recently, many genotyping protocols, supported by computational interpretative algorithms, have been developed for research and clinical purposes. In the real-life scenario, therapeutic management must take into consideration not only the disease and the history of the patient, but can also benefit from genotyping analyses. The Absorption, Distribution, Metabolism, and Excretion (ADME) pathways of drugs involve several genes, whose genotyping can guide treatment choice. For a long time, cancer treatment was based on cellular and histological differences of tumors. Increasing knowledge about genetic influences on ADME pathways permit the investigation of the relationship between genetic polymorphisms and drug reactions (adverse events, toxicity, safety, effectiveness) [10,11]. Several genes play a role in drug metabolization, the evaluation of genetic polymorphisms in target proteins and drug-metabolizing enzymes can support the development of individualized adjustments of drug dosage.

In summary, in this Special Issue, different studies reported the importance of genetic factors in individual response to pharmacological treatments. Three reviews focused on pharmacogenomics in oncology, psoriasis, and monogenic diabetes [6,7,8]. Furthermore, three research studies elucidate molecular aspects [9], genetic associations in large populations [5], and in selected ancestries [4].

Although the scientific literature provides several reports on the economic advantages derived from the pharmacogenomics test [12,13,14], to date, the routine use of pharmacogenomics testing is uncommon in many countries. Genetic analyses differ from routine laboratory tests, as they do not provide a numerical value and must be interpreted. Applied genomics requires new scientific skills, but in some contexts, there is still a considerable heterogeneity in the scientific background between geneticists who develop the tests and provide results and the clinicians who must use them in their clinical routine.

It is time to consider that translational medicine requires not only new knowledge but also training programs and clinical tools to bridge the gap between geneticists and clinicians, promoting the application of genomics at an individual level. In our perspective, the two priorities of pharmacogenomics are innovative education and new discoveries, probably in that order.

## Data Availability

Not applicable.
